# Location-Specific Disruption of the Body Schema in Chronic Low Back Pain

**DOI:** 10.1016/j.arrct.2026.100666

**Published:** 2026-07-01

**Authors:** Paul Teillet, Lucette Toussaint, Romain David, Rémi Cabirol, Maxime Billot, Romain Tisserand

**Affiliations:** a Université de Poitiers, Université de Tours, CNRS, CeRCA, Poitiers, France; b Université de Poitiers, ISAE-ENSMA, CNRS, PPRIME, Poitiers, France; c CIC Innovation Technologique INSERM 1402, Poitiers, France; d CHU de Poitiers, service de médecine physique et réadaptation, Poitiers, France

**Keywords:** Body image, Chronic pain, Low back pain, Pain measurement, Rehabilitation

## Abstract

**Objectives:**

To investigate whether chronic low back pain (CLBP) involves location-specific or more general body schema alterations, thereby helping to determine whether location-specific tasks may improve assessment of sensorimotor alterations and/or rehabilitation strategies for CLBP patients. And, secondarily, to examine whether pain intensity is associated with the extent of body schema alterations.

**Design:**

Between-subject comparative study with age- and sex-matched groups.

**Setting:**

This study was conducted at a University Hospital.

**Participants:**

Nineteen patients with CLBP and 19 healthy control participants matched for age and sex.

**Interventions:**

Not applicable.

**Main Outcome Measures:**

Body schema alterations were assessed using response time and accuracy of implicit motor imagery (IMI; ie, laterality judgment tasks) involving the painful location (low back) and a nonpainful location (hand). In CLBP patients, average pain intensity was assessed using numerical rating scales.

**Results:**

Compared with healthy adults, CLBP patients showed longer response time in the task involving the painful location (low back) but no difference in the task involving a nonpainful location (hand). No significant correlation was found between pain intensity and response time or accuracy.

**Conclusions:**

CLBP seems associated with location-specific alterations of the body schema rather than being a generalized sensorimotor impairment. Consequently, this study highlights the value of location-specific tasks as tools for objectively identifying pain-related sensorimotor alterations and their potential to improve rehabilitation.

Chronic low back pain (CLBP) is the leading musculoskeletal disorder in terms of years lived with disability worldwide and is considered chronic when lasting over 3 months.^1^ CLBP causes both location-specific and more general impairments in several sensorimotor functions, such as cortical reorganization related to trunk representation^2^ and changes in motor control strategies,^3^ respectively. However, it remains to be determined whether location-specific clinical treatments (ie, interventions specifically targeting the painful location), are relevant for CLBP patients.^4^ Future clarification of this issue could contribute to appropriately delineated rehabilitation programs for CLBP patients.

Sensorimotor alterations can be studied by investigating the body schema^5^ using implicit motor imagery (IMI), which illustrates how individuals integrate their body (or body parts) in movement planning and execution.^6^ IMI can be assessed by laterality judgment tasks,^7^ where individuals need to mentally maneuver their body (or body parts) to align with images of the body (or body parts) displayed on a screen, to determine whether the highlighted body part(s) correspond to the left or the right sides of the body. Disruption of peripheral sensory information, particularly by peripheral pain (ie, the extremities of the body), alters IMI performance.^8^ This suggests that alterations of sensorimotor information caused by pain in peripheral locations (eg, hands and feet) can impair the internal representation of these locations. By contrast, the effect of pain in axial locations (ie, along the central axis of the body) on their respective internal representations is unclear.^8^ As CLBP is considered as axial pain, it remains unclear whether CLBP impacts sensorimotor processes in a location-specific or in a more global manner.

A trunk laterality judgment task involving large-amplitude low back movements showed longer sensorimotor processing times in CLBP patients compared with controls,^9^ contrasting with previous studies reporting either reduced accuracy in CLBP patients^10^^,^^11^ or no difference,^12^ compared with healthy controls. This difference may arise from trunk movement amplitude suggested by the body position in the image. This amplitude was small in previous studies^10^, ^11^, ^12^ and large in another study.^9^ A large amplitude may encourage a more person-centered IMI strategy by limiting the use of visual matching strategies based on silent visual cues.^13^ Additionally, the study from Toussaint et al.^9^ was designed to investigate the effect of axial pain on IMI performance using a trunk laterality judgment task, and not to explore whether pain-related processes involve specific pain location or more general alteration of the body schema. This distinction remains critical, as previous studies comparing IMI performance between tasks involving painful (low back) and nonpainful locations (hand, foot) failed to reach a consensus: reduced accuracy in the CLBP group compared with healthy controls only for tasks involving the painful location,^11^ or no group differences for either location^12^ were found. Therefore, the question of whether CLBP-related sensorimotor alterations are location-specific or generalized remains unresolved. The relationship between pain intensity and IMI performance in CLBP has likewise remained inconsistent.^11^^,^^12^

Using the paradigm developed by Toussaint et al.,^9^ this study investigated whether CLBP involves location-specific or more general sensorimotor alterations. The objectives were (1) to determine the effect of CLBP on IMI performance in tasks involving either the specific painful location (a trunk laterality judgment task with large-amplitude movements of the low back) or a nonpainful location (a hand laterality judgment task), and (2) to explore the potential relationship between pain intensity and IMI performance. It was hypothesized that impaired performance would be observed in CLBP patients only for the task involving the specific painful location (low back), but not for the nonpainful location (hand). Additionally, it was hypothesized that greater pain intensity would correlate with poorer IMI performance.

## Methods

### Participants

Nineteen CLBP patients (table 1) were recruited from the University Hospital of Poitiers (“pain” group). Nineteen healthy control participants, age- and biological sex-matched (table 1), were recruited at the University of Poitiers (“control” group). The sample size was determined using G Power software, with an expected medium effect size of 0.25, an alpha level of 0.05 and statistical power of 0.90, resulting in a minimum required sample size of 19 participants. Eligibility criteria were as follows: ≥18 years (both groups); low back pain for more than 3 months (pain group); and no history of back pain (control group). Non-eligibility criteria were as follows: pain in locations other than low back (pain group), uncorrected visual disorders and previous participation in a similar task. The exclusion criterion was the inability to understand instructions.

**Table 1:** Participant characteristics

Participant Characteristics	Pain Group	Control Group
No. of participants	19 (11 women)	19 (11 women)
Age (y)	45.8±12.5	45.7±12.5
Age range (y)	20-69	22-68
Pain intensity		
7-day NRS (0-10)	6±2.1	N/A
Movement-related pain NRS (0-10)	6.2±1.7	N/A
Pain area		
Intense (cm²)	178±186	N/A
Very intense (cm²)	179±181	N/A
Full area (cm²)	551±293	N/A
Intense (%)	35±34	N/A
Very intense (%)	33±34	N/A

Abbreviations: NRS, numerical rating scale.

This study complied with the Declaration of Helsinki and was approved by the University ethic committee (IRB approval: CER-TP n° 2023-11-02). Participants’ written consent was obtained before the experiment.

### Measurements

#### Laterality judgment tasks

**Fig. 1:**
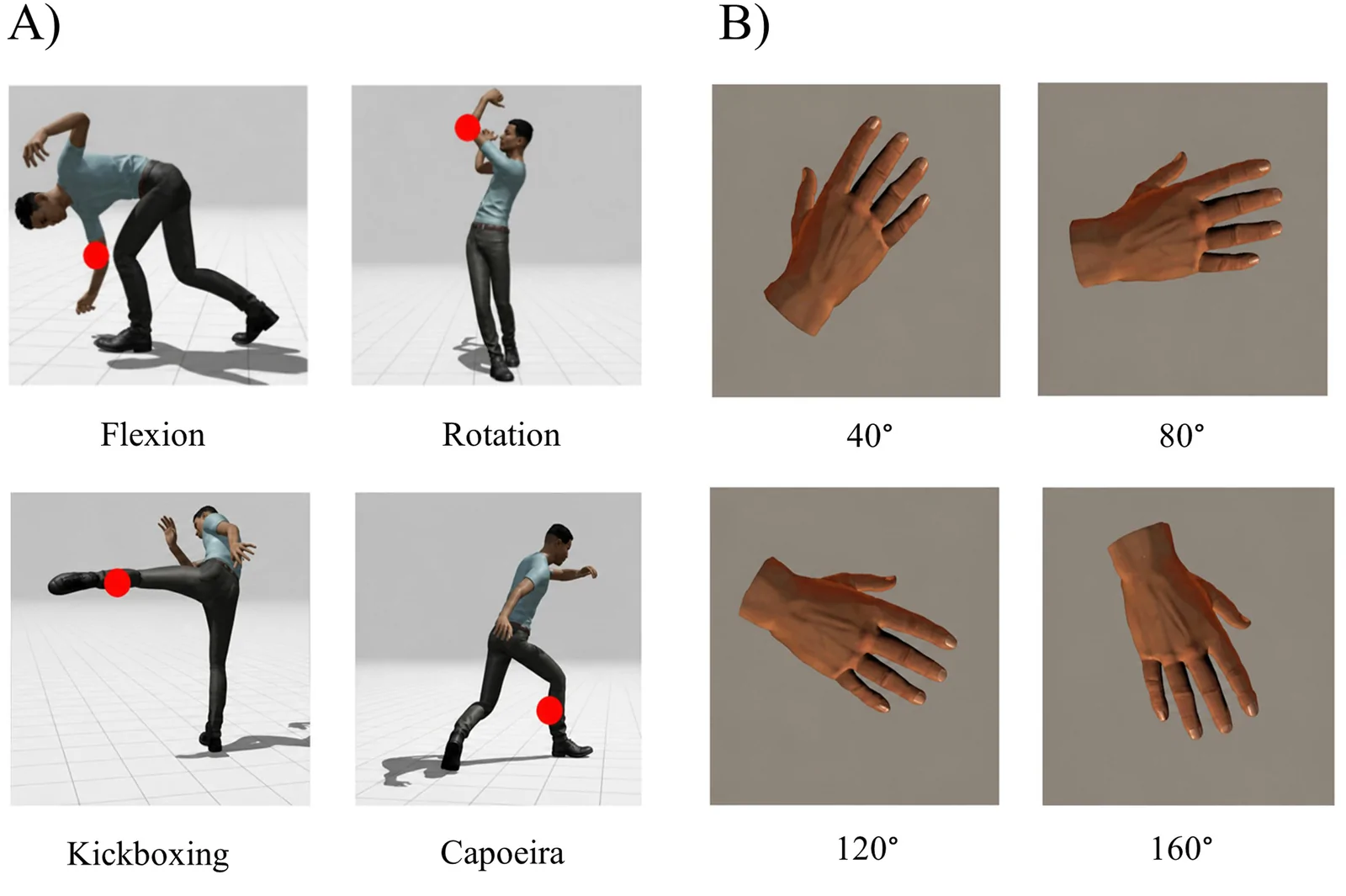
Illustration of the 4 movement types presented in the trunk laterality judgment task (panel A) and the 4 different hand orientations presented in the hand laterality judgment task (panel B).

Two computerized laterality judgment tasks assessed IMI, presented in a randomized order across participants. The first task, involving the painful location, randomly displayed 128 images of a human body avatar performing 4 large-amplitude trunk movements (flexion, axial rotation, kickboxing, capoeira) from 4 different viewpoints (back, right side, left side, front) (fig 1A). Participants were instructed to identify the laterality of the limb (arm or leg) where a red dot was located.^9^

The second task, not involving the painful location, randomly displayed 64 hand images from eight different rotations (40°, 80°, 120°, 160°, either clockwise or counter-clockwise) (fig 1B). Participants were instructed to identify the laterality of the hand displayed.^14^

Protocol procedure and participant positioning in front of the computer replicated the protocol detailed in the study by Toussaint et al.^9^ Each task took 5 to 10 minutes per participant.

#### Measures assessing pain intensity

Participants mapped their pain on a digital avatar using PRISMap software,^15^^,^^16^ categorizing intensity into 4 levels (light, moderate, intense, very intense). Pixel data were converted into cm² and the different intensities into percentages and were then collected for intense, very intense pain, and total pain area.

Pain intensity was also rated on a 0 to 10 numerical rating scale for both 7-day average and expected movement-related pain based on the 4 movement types displayed in the trunk task.

A multidimensional clinical response index (MCRI), integrating pain intensity, quality of life, functional disability, anxiety, depression, and pain distribution^17^ was also computed.

To prevent task-induced bias, all pain data were collected before the laterality judgment tasks.

### Statistical analysis

Accuracy (correct responses) and response time (milliseconds) were recorded using the E-Prime 2.0 software package (Psychology Software Tools Inc). Only correct response times were analyzed. Because data did not respect normality (Shapiro-Wilk) and variance homogeneity (Fischer-Scenedor), accuracy was arc-sinus-transformed and response time was log-transformed. For the trunk task, mixed ANOVAs were separately performed on accuracy and response time with the group as a between-participants factor and the movements and viewpoints as within-participants’ factors. For the hand task, mixed ANOVAs were conducted similarly, with the rotation as a within-participants factor. Significant interactions were analyzed by posthoc tests with Holm correction.

To avoid spurious effects from differences in accuracy-response time trade-offs, the inverse efficiency score (IES) was calculated,^18^, ^19^ combining accuracy and response time into a single score that reflects the average cognitive resource consumed.^20^ Lower values indicate more efficient processing. Group comparisons for the IES were conducted for each task using independent-samples Mann-Whitney tests.

Spearman’s rho correlations were performed between results from both laterality judgment tasks and pain intensity and the MCRI score. JASP software (0.16.3) was used for all analyses, with alpha set at .05.

## Results

Participant characteristics and pain-intensity outcomes are summarized in Table 1. Age did not significantly differ between groups (independent sample Student *t* test, *p*=0.969).

### Laterality judgment task results

#### Trunk laterality judgment task

**Fig. 2:**
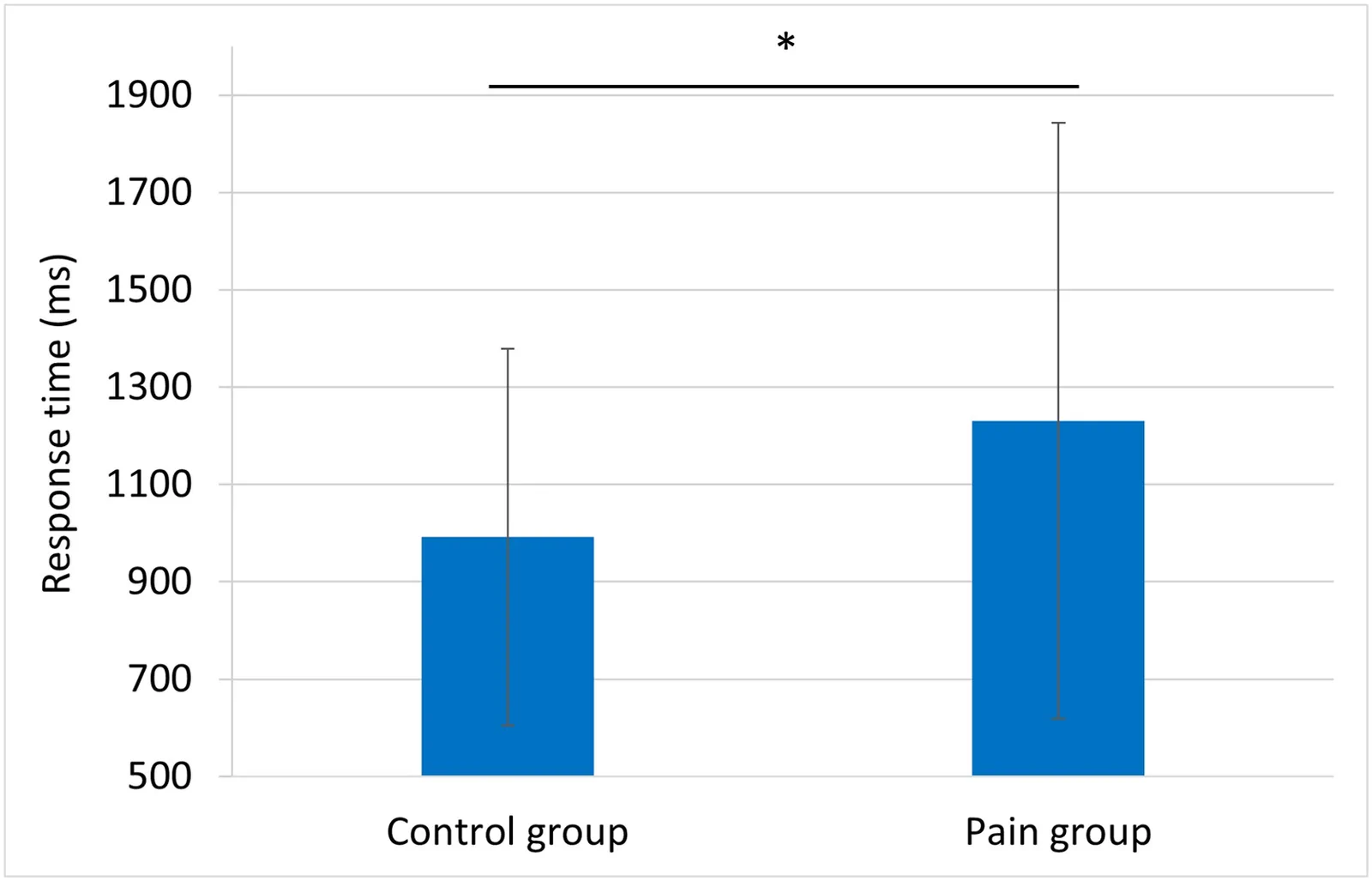
Average (±1 SD) response time (ms) in the trunk laterality judgment task in both control (n=19) and pain (n=19) groups.

**Fig. 3:**
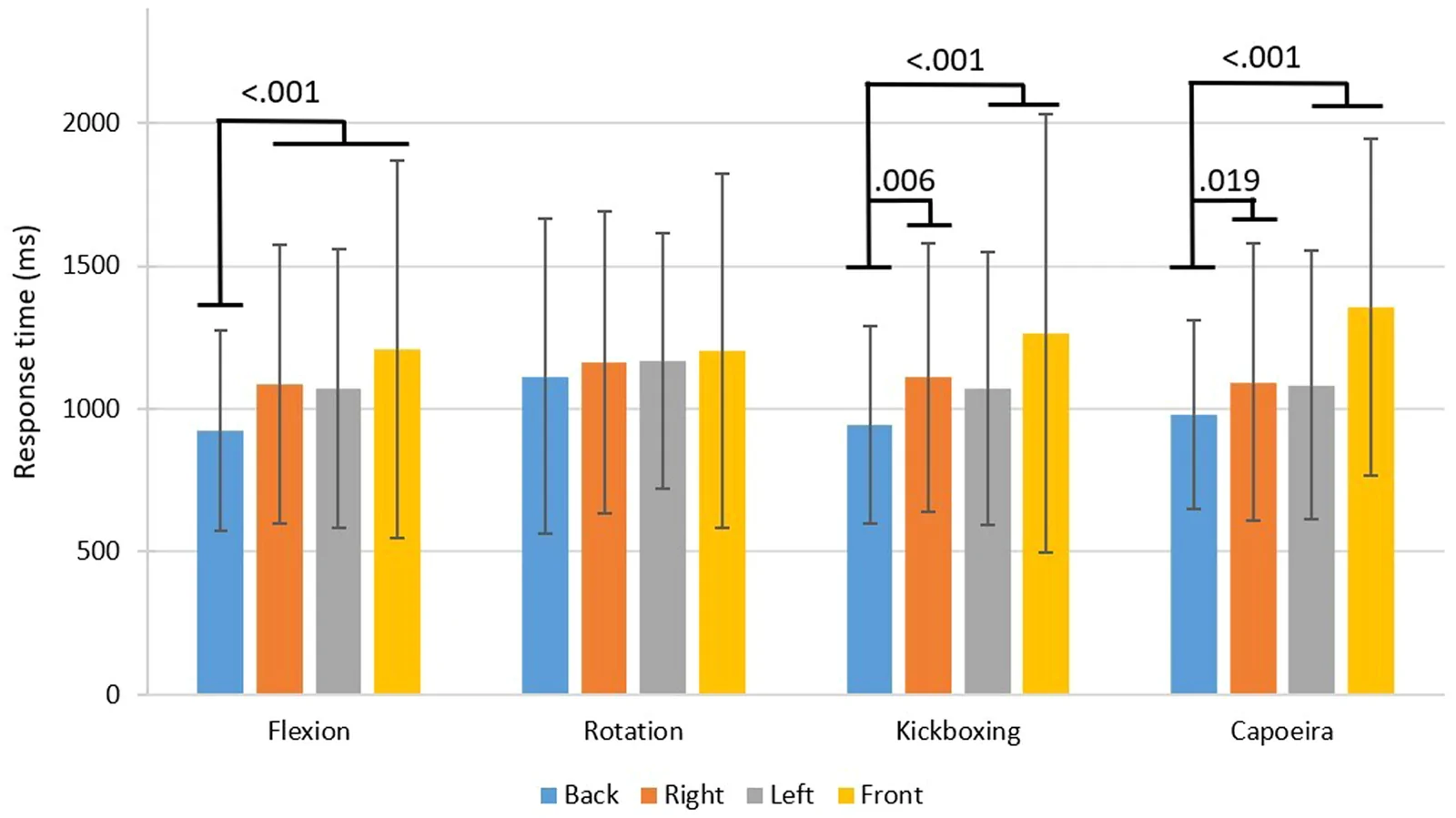
Average (±1 SD) response time (ms) relative to movement type and viewpoint in the trunk laterality judgment task for all participants (n=38).

For response time, the ANOVA revealed the main effects of group, movement, viewpoint, and a movement × viewpoint interaction (table 2). Results showed that the response time in the pain group was 239±225 ms longer on average compared to the control group (fig 2). For the movement × viewpoint interaction, post-hoc analyses demonstrated that average response time was shorter for the back viewpoint compared to the front, right, and left viewpoints for flexion (all *p*<0.001), kickboxing (all *p*<0.01), and capoeira movement (all *p*<0.05). There was no difference between viewpoints for the rotation movement (all *p*>0.05) (fig 3).

**Table 2:** ANOVA results for the trunk laterality judgment task

Response Time of the Trunk Laterality Judgment Task							
Factor	F	*P*	η^2^_p_	Interactions	F	*P*	η^2^_p_
Group	5.195	.029	0.097	Group × movement	0.986	.402	0.0009
Movement	11.435	<.001	0.011	Movement × viewpoint	10.892	<.001	0.017
Viewpoint	52.060	<.001	0.063	Viewpoint × group	0.775	.511	0.0009
				Group × movement × viewpoint	1.079	.378	0.002


Accuracy of the Trunk Laterality Judgment Task							
Factor	F	*P*	η^2^_p_	Interactions	F	*P*	η^2^_p_
Group	1.816	.186	0.006	Group × movement	0.968	.411	0.005
Movement	3.967	.010	0.021	Movement × viewpoint	3.471	<0.001	0.040
Viewpoint	5.118	.002	0.022	Viewpoint × group	0.811	.491	0.004
				Group × movement × viewpoint	1.149	.328	0.013

For accuracy, the ANOVA revealed the main effects of movement, viewpoint, and a movement × viewpoint interaction (table 2). Post-hoc analyses showed that, overall, accuracy for the front viewpoint was significantly reduced compared to the back viewpoint (decrease of 4%±9%, *p*<0.05). Accuracy in the front viewpoint for the capoeira movement (88%±32%) was significantly lower compared to the back viewpoint for the flexion (98%±13%, *p*<0.05) and kickboxing (99%±8%, *p*<0.05) movements. No significant interaction between movement and viewpoint was found. No significant difference was observed between groups (95%±21% for the control group and 97%±18% for the pain group, *p*>0.05).

#### Hand laterality judgment task

For response time, the ANOVA showed a main effect of viewpoint, without any effects of group or group × viewpoint interaction (table 3). Average response time for both groups was 1288±783 ms. Post-hoc analyses revealed shorter response times for the 40° viewpoint (979±407 ms) compared to the 80° viewpoint (1093±514 ms, *p*<0.001), both of them shorter than the 120° viewpoint (1326±688 ms, *p*<0.001), and all of them shorter than the 160° viewpoint (1824±1154 ms, *p*<0.001).

**Table 3:** ANOVA results for the hand laterality judgment task

Response Time of the Hand Laterality Judgment Task							
Factor	F	*P*	η^2^_p_	Interactions	F	*P*	η^2^_p_
Group	3.187	.083	0.053	Group *×* viewpoint	0.068	.977	0.0001
Viewpoint	114.751	<.001	0.264				


Accuracy of the Hand Laterality Judgment Task							
Factor	F	*P*	η^2^_p_	Interactions	F	*P*	**η**^2^_p_
Group	0.761	.389	0.008	Group *×* viewpoint	0.042	.988	0.0005
Viewpoint	12.983	<.001	0.169				

For accuracy, the ANOVA showed a main effect of viewpoint, without any effects of group or group × viewpoint interaction (table 3). The average accuracy for both groups was 94%±24%. Post-hoc analyses revealed decreased accuracy for the 160° viewpoint (86%±35%) compared to the 120° (95%±22%), 80° (97%±18%), and 40° (98%±16%) viewpoints (all *p*<0.01).

### Inverse efficiency score

For the trunk task, a significant group difference was found (U=112.0, *p*<0.05), with higher IES values in the pain group (1280±439 ms) than in the control group (1046±247 ms).

For the hand task, no significant difference was observed between groups (U=132.0, *p*=0.163), although the pain group showed numerically higher IES values (1522±636 ms) than the control group (1252±440 ms).

### Relationship between pain intensity or area and laterality judgment tasks

No significant correlation was found between IMI performance and pain intensity or area, nor with the MCRI (table 4).

**Table 4:** Relationship between pain intensity or area and laterality judgment tasks results. Rho corresponds to Spearman’s rho.

	Trunk Laterality Judgment Task				Hand Laterality Judgment Task			
	Response Time		Accuracy		Response Time		Accuracy	
	rho	*P*	rho	*P*	rho	*P*	rho	*P*
Pain intensity (n=19)								
7-day NRS	−0.20	.40	0.00	.98	0.21	.38	0.07	.77
Movement-related pain NRS	0.08	.73	−0.10	.67	0.37	.10	−0.14	.55
Pain area (n=19)								
Intense (cm²)	0.02	.90	0.16	.49	−0.10	.68	0.24	.30
Very intense (cm²)	−0.40	.08	−0.07	.77	−0.07	.77	−0.13	.59
Full area (cm²)	−0.38	.10	−0.14	.56	−0.31	.19	0.06	.80
Intense (%)	0.20	.40	0.24	.30	0.07	.75	0.29	.22
Very intense (%)	−0.32	.17	−0.11	.64	0.04	.86	−0.20	.39
MCRI (n=18)	0.19	.40	−0.27	.27	−0.17	.49	−0.46	.05

Abbreviations: NRS, numerical rating scale.

## Discussion

The objectives were (1) to determine the effect of CLBP on IMI performance in tasks involving either the specific painful location (low back) or a nonpainful location (hand), and (2) to explore the potential relationship between pain intensity and IMI performance. The main results showed that only in the task involving the painful location did CLBP patients have a longer response time than the control group. This finding suggests a location-specific alteration of the body schema, whereas no correlation was found between IMI performance and pain-intensity associated measures.

### The evidence of motor imagery engagement

Comparing response times between viewpoints is a reference methodology to assessment of IMI engagement.^7^ When comparing between different viewpoints, due to their requiring of the largest mental rotation, longer response times are expected in the viewpoints the furthest from the participant’s current orientation. In both tasks, response times increased with the amplitude of the required mental rotation. In the trunk task, response times were significantly longer for the front viewpoint than for the back viewpoint, reflecting the need for a 180° mental rotation to align their body perspective with the image (fig 3). In the hand task, a similar pattern was observed. Differences from previous studies^11^^,^^12^ may be partially explained by methodological variations. Here, the trunk task involved large-amplitude movements of the low back implying an IMI strategy, whereas previous studies used images with small amplitudes that may have led participants to rely on salient visual features rather than engaging in a person-centered strategy.^13^ Overall, these results strongly support that participants were involved in an IMI strategy mobilizing a person-centered mental rotation of the body.^7^^,^^9^^,^^14^^,^^21^

### Location-specific disruption of the body schema in CLBP

Consistent with the first hypothesis, CLBP patients showed reduced IMI performance, with longer response times (239±225 ms) than controls. This difference was observed only in the trunk task involving the painful body location (fig 2). No group difference was found for response time in the hand task. For accuracy, no group difference was found in either task. The IES confirmed a group difference in the trunk task only, with lower efficiency in CLBP patients. The longer response time and the absence of a difference in accuracy between groups in the trunk task replicate previous findings with a different group of CLBP patients.^9^ The preserved accuracy suggests that CLBP does not alter the integrity of cortical sensorimotor representation of the painful location, and that CLBP patients remain able to perform the task reliably.^8^ By contrast, the observed response time difference suggests an alteration in the processing speed of sensorimotor information.^8^ This contrasts with previous findings reporting reduced accuracy with preserved response time in CLBP patients.^11^ This discrepancy may be related to differences in task demands and instructions, with large-amplitude movements used in the present study potentially promoting a more effortful IMI strategy, resulting in longer response times while preserving accuracy. The fact that an alteration appears only in the trunk task supports the hypothesis of its being specifically related to the painful location. One explanation for the pattern of preserved accuracy and elongated response time observed in the trunk task is that chronic pain may lead to reduced movement of the painful location,^22^ driven by fear of movement aimed at limiting pain and preventing any risk of re-injury.^23^ This motor disengagement may limit the continuous updating of the body schema, as seen in immobilization studies.^14^^,^^24^ Together, these factors may contribute to a slowing of sensorimotor information processing for the painful location, but without fundamentally altering the accuracy of the internal body representation. This pattern suggests that axial pain impacts the body schema in a location-specific manner in CLBP patients, without affecting other non-painful locations such as the hands.

### Relationship between pain intensity and IMI performance

Contrary to the second hypothesis, no significant relationship was found between pain intensity and IMI performance which aligns with the findings of the study by Bray and Moseley.^11^ Although Linder et al.^12^ found a negative association between accuracy and 7-day pain intensity in CLBP patients, variance was relatively low (R² of 0.230) and should be interpreted with caution. The discrepancy with the current study may stem from methodological differences or other contributing factors such as the complex nature of pain. In particular, different pain phenotypes (eg, mechanical vs neuropathic) may differentially influence IMI performance, with mechanical pain associated with longer response times.^9^ Neither pain intensity nor the MCRI score showed any association with IMI performance, suggesting that it is not dependent on pain-related measures. This relationship may not be linear; and other factors could have a mediating role: cognitive functions,^25^ prior motor experience^26^ or fear of movement due to its neuromuscular control alterations,^27^ which may indirectly affect motor imagery by modifying the internal representation of movements. Overall, since no consensus currently exists on how pain intensity is related to IMI performance in CLBP patients, future studies are needed to refine the sensitivity of laterality judgment tasks.

### Study limitations

Pain assessment relied on subjective ratings prone to daily fluctuations,^28^, ^29^ potentially reducing their reliability relative to a single testing session. Furthermore, participants’ restricted range of pain intensity, mostly moderate, may have limited potential associations between pain-intensity measures and IMI performance. A wider distribution of pain intensities might reveal stronger associations, as far as higher pain intensity generally restricts movement.^30^ Evaluating movement-related pain may have been challenging due to some uncommon movements rarely experienced by participants (capoeira and kickboxing). Although full body images may have engaged more global body schema representations, the consistent involvement of large-amplitude movements of the painful location likely drove the observed performance. Furthermore, these images primarily emphasized lumbar movements, with limbs included to facilitate marker placement and visual discrimination.^9^ The relatively small sample size may have limited the statistical power to detect subtle effects. The cross-sectional design of the study precludes causal inference between pain and sensorimotor impairments. Lastly, patients were recruited from only one clinical center, which may limit the generalizability of the findings to a broader CLBP population.

### Clinical significance

Since response time was altered only in the trunk task, these findings suggest that sensorimotor alterations in CLBP are location-specific, extending observations from peripheral^31^^,^^32^ to axial pain. These findings may have potential clinical implications. Previous work has suggested that location-specific interventions targeting peripheral sensorimotor internal representations can decrease pain, using a graded motor imagery program among patients with complex regional pain syndrome.^32^ Such interventions involve short sessions (∼10 to 15 minutes) on a digital platform, which can be repeated several times a day over a period of several weeks. Although the efficacy of such programs in CLBP remains to be established, IMI-based laterality judgment tasks offer a simple, quick, and inexpensive tool to detect pain-related sensorimotor alterations. Further research is needed to determine their clinical utility in guiding rehabilitation strategies.

## Conclusions

This study supports a location-specific disruption of the body schema in CLBP, evidenced by longer sensorimotor processing times in the trunk task. This study also highlights the value of location-specific tasks as low-cost assessment tools that can be easily implemented in clinical routine to objectively identifying pain-related sensorimotor alterations and their potential to improve rehabilitation. However, no relationship was found between pain intensity and IMI performance, and further investigations is necessary.
